# Provision of Pediatric Immunization Services During the COVID-19 Pandemic: an Assessment of Capacity Among Pediatric Immunization Providers Participating in the Vaccines for Children Program — United States, May 2020

**DOI:** 10.15585/mmwr.mm6927a2

**Published:** 2020-07-10

**Authors:** Tara M. Vogt, Fan Zhang, Michelle Banks, Carla Black, Bayo Arthur, Yoonjae Kang, Paul Lucas, Brock Lamont

**Affiliations:** 1 CDC COVID-19 Emergency Response Team.

Recent reports suggest that routine childhood immunization coverage might have decreased during the coronavirus disease 2019 (COVID-19) pandemic (*1*,*2*). To assess the capacity of pediatric health care practices to provide immunization services to children during the pandemic, a survey of practices participating in the Vaccines for Children (VFC) program was conducted during May 12–20, 2020. Data were weighted to account for the sampling design; thus, all percentages reported are weighted. Among 1,933 responding practices, 1,727 (89.8%) were currently open; 1,397 (81.1%) of these reported offering immunization services to all of their patients. When asked whether the practice would likely be able to accommodate new patients to assist with provision of immunization services through August, 1,135 (59.1%) respondents answered affirmatively. These results suggest that health care providers appear to have the capacity to deliver routinely recommended childhood vaccines, allowing children to catch up on vaccines that might have been delayed as a result of COVID-19–related effects on the provision of or demand for routine well child care. Health care providers and immunization programs should educate parents on the need to return for well-child and immunization visits or refer patients to other practices, if they are unable to provide services (*3*).

The VFC program* is an entitlement program that provides federally purchased vaccines to eligible children aged ≤18 years at no cost. Approximately half of U.S. children are eligible to participate in the VFC program, mostly based on Medicaid enrollment or lack of insurance coverage, and an estimated 86% of U.S. pediatricians provide care in a VFC-enrolled practice (*4*,*5*). VFC provider practices include many types of health care providers; all serve at least some pediatric patients. Contact information for VFC program points of contact in VFC-enrolled practices and total number of federally purchased vaccine doses ordered are recorded in two CDC systems: the Provider Education and Assessment Reporting (PEAR) system^†^ and the Vaccine Tracking System (VTrckS).^§^ Using information from PEAR and VTrckS, 5,144 of the 37,949 (13.6%) practices enrolled in the VFC program as of May 6, 2020, were randomly sampled, with probability of selection proportional to the number of federally purchased vaccine doses shipped to the practice. A survey invitation that contained a link to a survey programmed using Research Electronic Data Capture software (version 9.5.13; Vanderbilt University) was emailed to VFC points of contact of the randomly selected practices during May 12–20; VFC points of contacts from 1,933 of the 5,144 practices (37.6%) responded from the 50 U.S. states, the District of Columbia, and Puerto Rico. To check for response bias, a follow-up assessment that involved conducting telephone calls to determine operational status among a random sample of 199 (6.2%) nonresponding provider practices was conducted. Survey responses were summarized overall and stratified by urban/rural location^¶^ and U.S. Census region.** Data were weighted to account for the sampling design, thus all percentages reported are weighted. Using SAS (version 9.4; SAS institute) and SUDAAN (version 11.0.1; Research Triangle Institute), statistical comparisons were made using chi-squared tests; a p-value of <0.05 was considered statistically significant. This investigation was determined by CDC to be public health surveillance. Therefore the CDC’s Institutional Review Board approval was not required.

Among 1,933 responding practices, 1,727 (89.8%) were currently open, and 206 (10.2%) were currently closed (including 197 [9.8%] that were temporarily closed; and nine [0.5%] that were permanently closed) (Table). Among open practices, 1,063 (61.7%) were offering reduced office hours for in-person visits. Practices in the Northeast were more likely to be closed (65, 15.0%) than were those in the South (42, 6.2%) and West (41, 10.0%). Reduced office hours for in-person visits were more common among urban practices (798, 63.7%) and those in the Northeast (263, 77.8%) than among rural practices (257, 55.4%) and those in all three other regions (53.8%–64.4%), respectively. Among 170 practices that were currently closed and excluding 36 “don’t know/not sure” responses, 131 (77.2%) reported that pediatric patients have been or will be referred to another medical home for immunization services.

**TABLE:** Operational status and provision of pediatric immunization services at practices, by health care provider characteristics – Vaccines for Children Provider Survey, May 2020

Characteristic	Total, no. (%)	Urban/Rural provider practice location,* no. (weighted %)			U.S. Census region,^†^ no. (weighted %)						
		Urban, reference	Rural	p-value^§^	Northeast, reference	Midwest	p-value^¶^	South	p-value^¶^	West	p-value^¶^
**Total**	**1,933 (100)**	**1,413 (73.7)**	**511 (26.3)**	**—**	**404 (20.7)**	**457 (23.6)**	**—**	**663 (34.8)**	**—**	**400 (20.9)**	**—**
**Current operational status of the practice in mid-May 2020 (n = 1,933)**											
Open	**1,727 (89.8)**	1,253 (89.2)	465 (91.4)	0.137	339 (85.0)	399 (87.5)	0.281	621 (93.9)	0.000	359 (90.0)	0.032
Closed	**206 (10.2)**	160 (10.9)	46 (8.6)		65 (15.0)	58 (12.5)		42 (6.2)		41 (10.0)	
**Among practices that are currently open, office hours for in-person visits, relative to prepandemic hours (n = 1,727)**											
Reduced	**1,063 (61.7)**	798 (63.7)	257 (55.4)	0.002	263 (77.8)	256 (64.4)	0.000	333 (53.8)	0.000	203 (56.4)	0.000
Not reduced	**664 (38.3)**	455 (36.3)	208 (44.6)		76 (22.2%)	143 (35.6)		288 (46.2)		156 (43.6)	
Among practices that are currently closed, pediatric patients have been or will be referred to a new medical home (n = 170)**											
Yes	**131 (77.2)**	101 (77.1)	30 (77.6)	0.950	35 (69.6)	36 (72.5)	0.753	25 (79.9)	0.316	35 (90.3)	0.024
No	**39 (22.8)**	30 (22.9)	9 (22.4)		16 (30.4)	13 (27.5)		6 (20.1)		4 (9.8)	
**Among practices that are currently open, offering routine immunization services to pediatric patients (n = 1,727)**											
All patients	**1,397 (81.1)**	1,012 (81.1)	378 (81.2)	0.013	261 (77.2)	312 (78.8)	0.177	522 (84.1)	0.014	295 (82.3)	0.238
A subset of patients	**254 (14.7)**	196 (15.5)	56 (12.3)		64 (19.0)	62 (15.3)		72 (11.6)		54 (15.1)	
No patients	**76 (4.2)**	45 (3.4)	31 (6.6)		14 (3.8)	25 (6.0)		27 (4.3)		10 (2.6)	
**Practice could likely provide immunization services to additional pediatric patients through the end of August (n = 1,933)**											
Yes	**1,135 (59.1)**	779 (55.5)	347 (68.4)	0.000	182 (45.5)	280 (61.0)	0.000	422 (64.1)	0.000	242 (61.2)	0.000
No^††^	**418 (21.3)**	334 (23.4)	84 (15.9)		128 (31.2)	85 (18.8)		121 (18.0)		84 (20.5)	
Don’t know/Not sure	**380 (19.6)**	300 (21.1)	80 (15.7)		94 (23.3)	92 (20.3)		120 (17.9)		74 (18.3)	

* Classification of urban (metropolitan) or rural (nonmetropolitan) was based on county of practice location using the 2013 National Center for Health Statistics Urban–Rural Classification Scheme for Counties (https://www.cdc.gov/nchs/data/series/sr_02/sr02_166.pdf). Practices in Puerto Rico (nine) are not shown.

^†^ https://www2.census.gov/geo/docs/maps-data/maps/reg_div.txt. Practices in Puerto Rico (nine) are not shown.

^§^ Chi-squared test, compared with urban location.

^¶^ Chi-squared test, compared with Northeast region.

** Among 206 practices reporting currently closed, those that answered “Don’t know/Not sure” to their pediatric patients having been or will be referred to a new medical home (36) are not shown.

^††^ Includes practices that are currently open or planning to reopen but reported not likely being able to accept additional patients (400), practices permanently closed (nine), and practices not resuming immunization services for all patients (nine).

Among 1,727 open practices, 1,397 (81.1%) reported currently offering immunization services to all their pediatric patients, 254 (14.7%) to some pediatric patients, and 76 (4.2%) to no pediatric patients. A majority of practices currently offering immunization services to some children reported offering them to children aged <12 months (224, 89.2%) and 1–2 years (204, 81.4%), whereas less than half reported offering services to children aged 3–6 years, 7–10 years, or 11–18 years (Figure).

**FIGURE:**
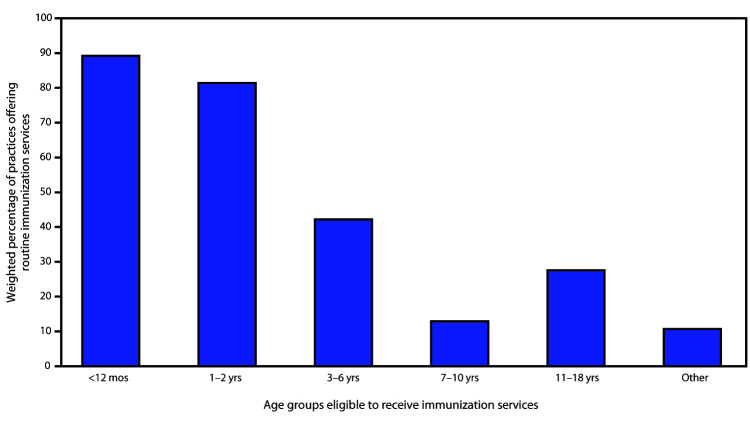
Pediatric age groups* eligible to receive routine immunization services at 254 practices not offering immunization services to all pediatric patients — United States, May 2020 * Categories are not mutually exclusive. "Other" includes age categories not reflected in the survey options (e.g., newborns only), patients with medical conditions or risk factors, and other scenarios such as patients behind on immunizations or parental request for vaccination.

Among all 1,933 providers participating in the survey, 1,397 (72.8%) reported currently offering immunization services to all pediatric patients; 344 (17.7%) reported that they would be offering immunizations to all children by July 1; 174 (8.7%) at some date after July 1; and 18 (0.8%) reported that the practice will not resume providing immunization services to all patients. Nine of these 18 practices reported being permanently closed, and nine would not resume immunization services to all patients for other reasons. When asked whether the practice would likely be able to accommodate new patients for immunization services through August, 1,135 (59.1%) of the 1,933 practices answered affirmatively, 418 (21.3%) either responded that this was not likely or the practice was permanently closed or not resuming immunization services for all patients, and 380 (19.6%) responded that they were unsure; urban practices and those in the Northeast were less likely to be able to accommodate new patients compared with rural practices and those in the other three regions (Table). The assessment of a random sample of 199 (6.2%) of 3,211 nonresponding practices found that 20 (10.1%) were currently closed or had unknown operational status, similar to survey respondents.

## Discussion

Ensuring that immunization services are maintained or reinitiated is essential for protecting persons and communities from vaccine-preventable diseases and outbreaks during the COVID-19 pandemic. However, notable declines in pediatric vaccine doses ordered and administered were observed beginning in March (*1*,*2*), and a survey of New York City preventive health care provider practices in April found that many have reduced or might soon reduce hours of operation, or temporarily or permanently close for a variety of reasons related to the pandemic (*6*). The results of the current national survey indicate that a majority of VFC-enrolled practices were open and offering routine immunization services to all pediatric patients in May or anticipate doing so in the near future. Further, over half of the practices were likely able to accommodate new patients over the coming months, which should help those families seeking immunization services because their routine health care provider practice is closed. In addition, after a sharp decline in VFC vaccine orders beginning in March and continuing through April (*1*), orders during the second half of May 2020 and the first 3 weeks of June were relatively comparable to those from the same period in 2019 (J Santoli, CDC, unpublished data, 2020), suggesting that the current immunization infrastructure can meet the expected need to provide vaccines that are overdue to many children.

Results from the survey did, however, raise concerns about access to routine immunization services among certain populations of children, particularly those living in urban areas and in the Northeast. Practices in these areas were more likely to report offering reduced in-person office hours and less likely to be able to accommodate new patients for immunization services through August, compared with providers in rural areas and in other regions. If the number of VFC-eligible children increases in these areas as a result of loss of health insurance because of pandemic-related unemployment (*7*), children whose medical home is not a VFC-enrolled practice will need to seek immunization services from a practice that is VFC-enrolled; a shortage of local VFC-enrolled practices willing and able to accommodate such patients could result in declines in coverage. Results also indicate that practices are prioritizing offering vaccination to younger children, consistent with CDC guidance emphasizing the importance of ongoing delivery of well child care, prioritizing children up to age 24 months, followed by young children, and then extending through adolescence (*3*). However, catch-up vaccination of school-aged children is also important throughout the summer to ensure children are fully vaccinated and able to meet school vaccination requirements before the commencement of the 2020–21 school year.

The findings in this report are subject to at least three limitations. First, the sample was limited to practices enrolled in the VFC program, and results might not be generalizable to practices that do not administer pediatric vaccines through the VFC program. Second, the survey’s response rate was approximately 38%, and nonresponse could be related to operational status, because staff members in practices that are permanently or temporarily closed might not have received the survey. However, the follow-up assessment to determine operational status among a random sample of nonresponding practices indicated that this was unlikely. In addition, practice size was not collected from survey respondents, precluding an assessment of associations between practice size and operational status and capacity to provide immunization services. Finally, because the size of the pediatric population requiring catch-up vaccination is currently unknown, the impact of findings on provider practice capacity cannot be quantified.

With the number of VFC-eligible children expected to increase as a result of the COVID-19 pandemic, it is important that CDC, state, and local public health departments, as well as other immunization partners, ensure that parents of newly VFC-eligible children are aware of the availability of publicly funded vaccine through the VFC program (*8*). To facilitate catch-up vaccination, these entities must educate parents and caregivers, using culturally appropriate approaches, about the importance of resuming immunization and other well child care visits that might have been missed during the early stage of the COVID-19 pandemic, while reassuring them that these visits can be done safely during the pandemic as health care providers take steps to reduce the risk of SARS-CoV-2 transmission (*3*,*9*,*10*).^††^ In addition, health care providers should consider reaching out to their patients about the importance of well-child visits and should use their systems (e.g., state immunization information system and electronic health records) to identify patients who are overdue for vaccines and conduct recall activities to schedule appointments as soon as possible.^§§^ Providers might also consider applying for the CARES Act Provider Relief Fund to receive financial assistance to offset financial losses related to the pandemic.^¶¶^ Resumption of vaccination activities is critical to protecting children and adolescents from vaccine-preventable diseases as well as to preventing outbreaks. As considerations about reopening schools in the fall continue, state and local immunization programs should work with local health care providers to facilitate catch-up vaccination activities to ensure student compliance with state and local vaccination requirements.

**Summary**

**What is already known about this topic?**

Declines in routine childhood immunization coverage have been reported during the COVID-19 pandemic.

**What is added by this report?**

A May 2020 survey of 1,933 practices participating in the Vaccines for Children program found that 1,727 (89.8%) were currently open, including 1,397 (81.1%) offering immunization services to all pediatric patients. Among responding practices, 1,135 (59.1%) were likely able to provide immunization services to new pediatric patients if necessary.

**What are the implications for public health practice?**

Practices appear to have the capacity to deliver routinely recommended vaccines, allowing children who have missed vaccine doses because of the pandemic to catch up. Practices that are unable to provide immunization services should refer patients to other practices.

## Footnotes

* Children aged ≤18 years are eligible to receive vaccine through the Vaccines for Children Program if they are Medicaid-eligible, uninsured, American Indian/Alaska Native, or underinsured and vaccinated at federally qualified health centers, rural health clinics, or provider sites with an approved deputization agreement with the state public health department (https://www.cdc.gov/vaccines/programs/vfc/index.html).

† The PEAR system is an online quality assurance tool developed to improve VFC Program oversight and document compliance with VFC regulations during provider site visits.

§ VTrckS is a secure, web-based information technology system that integrates the entire publicly funded vaccine supply chain from purchasing and ordering through distribution to participating state, local, and territorial health departments (referred to as “awardees”) and health care providers.

** https://www2.census.gov/geo/docs/maps-data/maps/reg_div.txt.

††
https://www.cdc.gov/vaccines/pandemic-guidance/ .

§§
https://www.aap.org/en-us/about-the-aap/aap-press-room/campaigns/call-your-pediatrician/Pages/default.aspx .

¶¶
https://www.hhs.gov/coronavirus/cares-act-provider-relief-fund/index.html .
